# Signal transduction in l-DOPA-induced dyskinesia: from receptor sensitization to abnormal gene expression

**DOI:** 10.1007/s00702-018-1847-7

**Published:** 2018-02-02

**Authors:** Giada Spigolon, Gilberto Fisone

**Affiliations:** Department of Neuroscience, Karolinska Institutet, Retzius väg 8, 17177 Stockholm, Sweden

**Keywords:** Dopamine receptors, cAMP-dependent protein kinase, Dopamine- and cAMP-regulated phosphoprotein of 32 kDa, Extracellular signal-regulated kinases 1 and 2, Mammalian target of rapamycin, Gene transcription

## Abstract

A large number of signaling abnormalities have been implicated in the emergence and expression of l-DOPA-induced dyskinesia (LID). The primary cause for many of these changes is the development of sensitization at dopamine receptors located on striatal projection neurons (SPN). This initial priming, which is particularly evident at the level of dopamine D1 receptors (D1R), can be viewed as a homeostatic response to dopamine depletion and is further exacerbated by chronic administration of l-DOPA, through a variety of mechanisms affecting various components of the G-protein-coupled receptor machinery. Sensitization of dopamine receptors in combination with pulsatile administration of l-DOPA leads to intermittent and coordinated hyperactivation of signal transduction cascades, ultimately resulting in long-term modifications of gene expression and protein synthesis. A detailed mapping of these pathological changes and of their involvement in LID has been produced during the last decade. According to this emerging picture, activation of sensitized D1R results in the stimulation of cAMP-dependent protein kinase and of the dopamine- and cAMP-regulated phosphoprotein of 32 kDa. This, in turn, activates the extracellular signal-regulated kinases 1 and 2 (ERK), leading to chromatin remodeling and aberrant gene transcription. Dysregulated ERK results also in the stimulation of the mammalian target of rapamycin complex 1, which promotes protein synthesis. Enhanced levels of multiple effector targets, including several transcription factors have been implicated in LID and associated changes in synaptic plasticity and morphology. This article provides an overview of the intracellular modifications occurring in SPN and associated with LID.

## Introduction

The last two decades have witnessed a remarkable progress in the understanding of the mechanisms mediating the effects of dopamine replacement therapies in Parkinson’s disease (PD). This is particularly evident with regard to l-DOPA, which is still the most effective therapy to control the motor symptoms of PD. A number of molecular changes produced in response to administration of l-DOPA have been identified and many of them have been tested for their involvement in the development and expression of dyskinesia. Most of this information has been obtained from studies centered on the striatum, which is the main component of the basal ganglia and the principal target of anti-parkinsonian medications. This article provides an overview of the signaling abnormalities implicated in l-DOPA-induced dyskinesia (LID), with special emphasis on postsynaptic mechanisms occurring in striatal neurons.

## Striatal projection neurons and control of motor function

In PD, the effects of l-DOPA are particularly evident at the level of the GABAergic projection neurons that connect the striatum to the efferent nuclei of the basal ganglia (internal segment of the globus pallidus and substantia nigra pars reticulata). Striatal projection neurons (SPN) express high levels of two major classes of dopamine receptors: the dopamine D1 receptors (D1R), whose activation increases the production of cAMP, and the dopamine D2 receptors (D2R), which inhibit cAMP synthesis. The opposite actions of these receptors on cAMP depend on their coupling to distinct G-proteins. Thus, D1R activate a Gαolf protein that stimulates the activity of adenylyl cyclase (AC), whereas D2R are coupled to Gαi/o-mediated inhibition of AC (Herve et al. 1993; Stoof and Kebabian 1981; Zhuang et al. 2000).

A fundamental feature of the basal ganglia circuitry is that striatal D1R and D2R are for the most part segregated into two separate populations of SPN, which innervate directly, or indirectly the output structures of the basal ganglia. This difference in connectivity confers to the SPN of the direct pathway (dSPN) the ability to promote motor activity, whereas the SPN of the indirect pathway (iSPN) suppresses motor activity. In addition, the selective expression of D1R in dSPN, and of D2R in iSPN, confers to dopamine the ability to enhance excitability of dSPN and to reduce excitability of iSPN, thereby promoting motor activity (Albin et al. 1989; DeLong 1990; Gerfen 1992).

## Dopamine receptor sensitization: a fundamental step toward the development of dyskinesia

A major causal factor at the basis of LID is the development of sensitization at striatal dopamine receptors. This phenomenon, which can be viewed as a homeostatic response to the progressive loss of dopamine input to the basal ganglia, has been characterized in particular detail at the level of the D1R located on dSPN, but is not necessarily limited to this neuronal population.

**Fig. 1:**
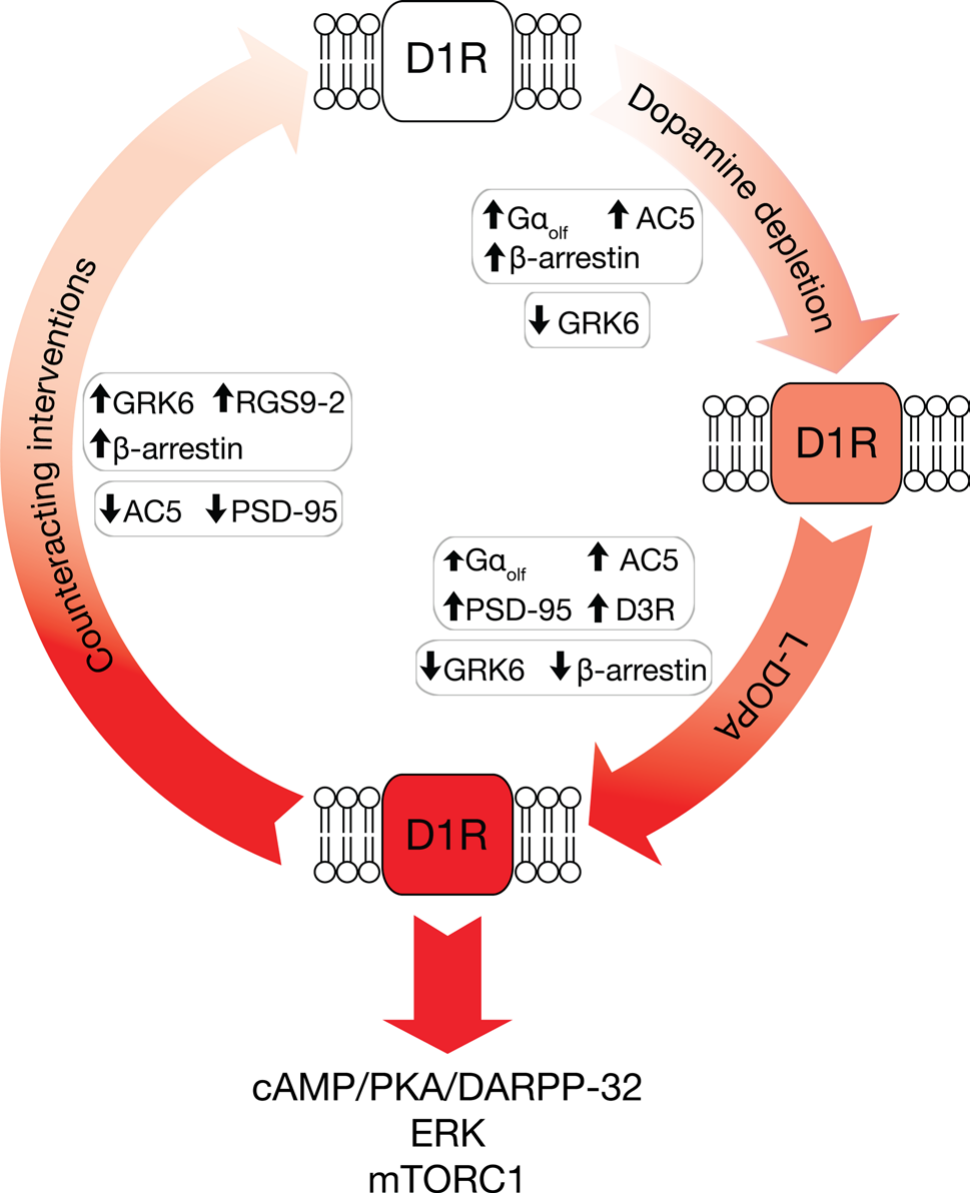
Schematic diagram illustrating the main mechanisms implicated in the sensitization of D1R associated with LID. In PD, the loss of dopamine input to the striatum increases the expression of the Gαolf protein and adenylyl cyclase type 5 (AC5). This change enhances the ability of D1R, which are expressed in the striatal projection neurons of the direct pathway (dSPN), to stimulate the synthesis of cAMP, a primary mediator of dopamine transmission. The increases in Gαolf protein and AC5 are paralleled by reduced expression of the G-protein-coupled receptor kinase 6 (GRK6), which decreases D1R phosphorylation, β-arrestin binding and receptor internalization. Dopamine depletion is also accompanied by increased expression of β-arrestin, which may counteract D1R sensitization. However, this effect is reversed by administration of l-DOPA. In contrast, overexpression of AC5 and, at least in part, Gαolf protein, as well as downregulation of GRK6, persists in dSPN even following administration of l-DOPA. Importantly, treatment with l-DOPA increases the levels of the postsynaptic density 95 (PSD-95) and dopamine D3 receptors (D3R). This further reinforces D1R-mediated transmission, leading to activation of the cAMP/PKA/DARPP-32, ERK and mTORC1 signaling cascades. The importance of these mechanisms is underscored by several studies, indicating that overexpression of GRK6 and β-arrestin, or downregulation of AC5 and PSD-95, decrease dyskinesia. In line with these findings, and further supporting the crucial role played by receptor sensitization in LID, this condition is also reduced by overexpression of the regulator of G-protein signaling 9-2 (RGS9-2), or by combining the administration of l-DOPA with D3R antagonists

Initial studies in toxin-based models of PD did not detect any increase in the number and affinity of striatal D1R (Aubert et al. 2005; Breese et al. 1987; Joyce 1991; Marshall et al. 1989; Savasta et al. 1988). This conclusion was supported by clinical studies in post-mortem samples from Parkinsonian patients (Hurley et al. 2001; Pimoule et al. 1985; Shinotoh et al. 1993). However, more recent investigations have identified a number of complex modifications affecting D1R and their associated signaling machinery, which prime this system to respond abnormally when exposed to dopaminergic drugs. This *original priming* is reinforced and maintained by additional molecular alterations produced by chronic administration of l-DOPA, which constitutes a *secondary priming* event (Fig. 1).

## Abnormal D1R trafficking and cell surface localization

Studies in non-human primates and rodents have shown that LID is associated with accumulation of D1R at the cell surface and that this effect is caused by disrupted receptor internalization, rather than overexpression (Aubert et al. 2005; Berthet et al. 2009; Guigoni et al. 2007). Notably, l-DOPA-mediated activation of D1R has been shown to impair the ubiquitin–proteasome system, thereby contributing to maintain D1R at the plasma membrane (Berthet et al. 2012). This phenomenon has been associated with the disassembling and reduced catalytic activity of the 26S proteasome (Barroso-Chinea et al. 2015).

The abnormal D1R trafficking implicated in LID has also been attributed to increased striatal levels of the postsynaptic density 95 (PSD-95), a member of the membrane-associated guanylate kinase proteins (Nash et al. 2005; Porras et al. 2012). PSD-95 acts as a postsynaptic scaffold, interacting with both D1R and N-methyl-D-aspartate (NMDA) glutamate receptors (Zhang et al. 2009; Fiorentini et al. 2003) (see article by Mellone and Gardoni in this issue for a detailed description of the involvement of glutamate receptors in dyskinesia). In experimental models of PD, lentiviral-mediated downregulation of PSD-95 enhances the lateral diffusion and internalization of D1R, leading to reduced receptor content at the cell surface. Notably, the same intervention diminishes the severity of LID (Porras et al. 2012).

The sensitized response of D1R to l-DOPA is associated with increased expression of dopamine D3 receptors in dSPN (Bezard et al. 2003; Bordet et al. 1997; Guillin et al. 2001). This event has significant functional repercussions, since the formation of heteromers with dopamine D3 receptors strengthens D1R transmission (Fiorentini et al. 2008; Marcellino et al. 2008). Thus, the up-regulation of dopamine D3 receptors may represent a parallel pathological mechanism, which further exacerbates D1R sensitization, contributing to the development of LID. In line with this possibility, several antagonists at dopamine D3 receptors have been shown to reduce the dyskinetic response to l-DOPA (Bezard et al. 2003; Kumar et al. 2009; Visanji et al. 2009; but see also Mela et al. 2010).

## Controlling D1R sensitized transmission and LID via G-protein coupled receptor kinases

The impact on LID of abnormal recruitment of D1R at the cell surface is highlighted by a series of studies based on the overexpression of the G-protein-coupled receptor kinase (GRK) 6. GRK6 is highly expressed in the striatum and involved in the desensitization of D1R (Ahmed et al. 2008; Li et al. 2015). Activated D1R are phosphorylated by GRK6 (Li et al. 2015), which leads to binding of β-arrestin and internalization (Ferguson et al. 1996). In the rat 6-hydroxydopamine (6-OHDA) model of PD, GRK6 expression is reduced (an effect which persists even after administration of l-DOPA) (Ahmed et al. 2008), suggesting a possible involvement in D1R sensitization. In support of this possibility, overexpression of GRK6 by lentiviral delivery promotes D1R internalization, thereby normalizing cell surface expression and counteracting sensitized transmission (Ahmed et al. 2010, 2015a). Importantly, enhanced expression of GRK6 has been shown to reduce LID in rodent and non-human primate models of PD (Ahmed et al. 2010).

An analogous approach to control D1R sensitization and LID is based on targeting β-arrestin. The potential efficacy of this intervention was initially suggested by the observation that dopamine depletion increases the levels of β-arrestin2 in the brain of 1-methyl-4-phenyl 1,2,3,6-tetrahydropyridine (MPTP)-treated monkeys and that this effect is reversed by l-DOPA (Bezard et al. 2005; Price et al. 2010). Subsequent studies performed in several models of PD and LID have shown that overexpression of β-arrestin2 reduces some of the biochemical markers associated with LID and attenuates this condition. In contrast, deletion of β-arrestin2 enhances the dyskinetic effect of l-DOPA (Urs et al. 2015).

Similarly to GRK6, also GRK3 is highly expressed in the striatum and down-regulated in response to dopamine depletion (Ahmed et al. 2008). Interestingly, GRK3 has been found to reduce LID independently of its ability to phosphorylate dopamine receptors and initiate their internalization (Ahmed et al. 2015b). The effect of GRK3 is instead mediated via its regulator of G-protein signaling (RGS) homology domain, which binds to and sequesters active Gαq/11 protein (Carman et al. 1999). This G-protein couples receptors to stimulation of protein kinase C and calcium/calmodulin protein kinase II (Hepler and Gilman 1992), which are, therefore, inhibited by overexpression of GRK3. The downstream mechanism at the basis of the ability of GRK3 overexpression to reduce dyskinesia is not yet clear, but it has been proposed that reduced expression of the transcription factor ∆FosB, which is implicated in LID (see below), may be involved (Ahmed et al. 2015b).

## Gαolf protein dysregulation in LID

D1R-mediated signal transduction is linked to the activation of a Gαolf protein positively coupled to AC (Herve et al. 2001; Zhuang et al. 2000). Several studies examined the involvement of this G-protein in the sensitization of D1R associated with PD. Work with experimental models and post-mortem samples from PD patients showed that the loss of striatal dopamine leads to up-regulation of Gαolf protein (Corvol et al. 2004; Herve et al. 1993; Penit-Soria et al. 1997; Marcotte et al. 1994; Alcacer et al. 2012; Rangel-Barajas et al. 2011; Ruiz-DeDiego et al. 2015b; Morigaki et al. 2017). It was also shown that this effect subsides in response to administration of l-DOPA (Corvol et al. 2004; Rangel-Barajas et al. 2011). However, in a recent study Morigaki et al. (2017) show that, in 6-OHDA lesion mice, the levels of Gαolf protein coupled to D1R, although reduced by l-DOPA, remain elevated in comparison with naïve mice. The same authors also show that a different regulation occurs in D2R-expressing iSPN, where the Gαolf protein is coupled to adenosine A2A receptors. In this case, administration of l-DOPA to PD mice down-regulates the Gαolf protein below the levels observed in naïve mice. This, in turn, promotes D2R transmission, which is antagonized by tonic activation of A2A receptors (Schiffmann et al. 2007). Thus, the overall effect of these distinct, cell-specific regulations of the Gαolf protein is to potentiate the effects of l-DOPA and dopamine receptor agonists on both D1R- and D2R-mediated transmission.

In spite of these findings, it has been reported that partial genetic inactivation of the *Gnal* gene, which fully counteracts the up-regulation of Gαolf protein associated with dopamine depletion, does not modify LID (Alcacer et al. 2012). Further studies will be necessary to characterize the potential impact of the Gαolf protein on dyskinesia. For instance, based on its different regulation in D1R- and D2R-expressing SPN (Morigaki et al. 2017), it would be interesting to examine the effects of selective downregulation of *Gnal* in dSPN and iSPN.

## Attenuating sensitization and dyskinesia via RGS proteins

RGS proteins typically act by promoting the GTPase activity of Gα-proteins, thereby hastening their inactivation (Ross and Wilkie 2000). The RGS9-2 protein is particularly abundant in dopamine-innervated regions, such as the striatum (Rahman et al. 1999), where it reduces dopamine receptor-mediated motor responses (Rahman et al. 2003). In the MPTP non-human primate model of PD, viral-vector-mediated overexpression of RGS9-2 diminishes LID, an effect which has been proposed to depend on reduced sensitization and normalization of transmission at both D1R and D2R (Gold et al. 2007).

The RGS4 is expressed in both dSPN and iSPN (Taymans et al. 2004). Chronic administration of l-DOPA increases RGS4 mRNA in the sensorimotor striatum, and this effect positively correlates with the severity of dyskinetic behavior (Ko et al. 2014). Furthermore, intracerebral infusion of antisense oligonucleotides against RGS4, in parallel with repeated administration of l-DOPA, reduces the development of dyskinesia. Therefore, the way by which RGS4 modulates LID differs from that of RGS9-2 and remains to be fully clarified. In this regard, a modification of the interaction between RGS4 and the metabotropic glutamate type 5 receptor (mGlu5R) has been proposed as a potential mechanism (Ko et al. 2014).

## Role of adenylyl cyclase type 5 in LID

In the striatum, the Gαolf protein couples D1R to stimulation of AC type 5 (AC5), which is highly expressed in SPN (Glatt and Snyder 1993; Mons and Cooper 1994). Work performed in the 6-OHDA rat model of PD shows that dopamine depletion leads to overexpression of AC5, which persists even after repeated administration of l-DOPA and correlates with the severity of dyskinesia (Rangel-Barajas et al. 2011). Up-regulation of AC5 is causally linked to LID. Thus, knockout, or lentiviral-mediated downregulation of AC5 in the dorsal striatum were found to attenuate LID in a PD mouse model (Park et al. 2014).

## From sensitized D1R to abnormal cAMP signaling

As outlined above, activation of striatal D1R leads to Gαolf-mediated stimulation of AC5 and increased synthesis of cAMP. The sensitization caused by dopamine depletion strongly enhances the ability of l-DOPA to promote this response, thereby hyper-activating cAMP-dependent protein kinase (PKA). This effect is implicated in LID, which is counteracted by administration of the PKA inhibitor, Rp-cAMPS (Lebel et al. 2010). In SPN, cAMP signaling depends on the dopamine- and cAMP-regulated phosphoprotein of 32 kDa (DARPP-32), which amplifies responses produced by stimulation of PKA through inhibition of protein phosphatase-1 (PP-1) (Fienberg et al. 1998; Greengard 2001). Abnormal activation of DARPP-32, which is mediated by PKA-dependent phosphorylation at Thr34, has been described in rodents and non-human primate models of LID (Picconi et al. 2003; Santini et al. 2007, 2010). In line with these observations, DARPP-32-deficient mice, or mice expressing a mutated form of DARPP-32 lacking Thr34, are less prone to develop LID (Santini et al. 2007, 2012). Attenuated LID has also been described in mice lacking DARPP-32 specifically in the D1R-enriched dSPN (Bateup et al. 2010). In contrast, genetic inactivation of DARPP-32 in iSPN does not modify the dyskinetic response to l-DOPA (Bateup et al. 2010).

The dysregulation of the PKA/DARPP-32 signaling cascade associated with LID is linked to impaired synaptic plasticity. In particular, abnormal PKA-mediated phosphorylation of DARPP-32, leading to suppression of PP-1 activity, has been implicated in the loss of depotentiation (i.e. in the inability to revert long-term potentiation; LTP) at glutamatergic corticostriatal terminals (Picconi et al. 2003). The absence of bidirectional control of synaptic plasticity is a general feature of dyskinesia and has been shown to occur at both dSPN and iSPN (Calabresi et al. 2016; Picconi et al. 2003; Thiele et al. 2014) (see article by Picconi et al. in this issue for a detailed description of the alterations of synaptic plasticity in LID).

## Extracellular signal-regulated kinases 1/2: central players in LID

**Fig. 2:**
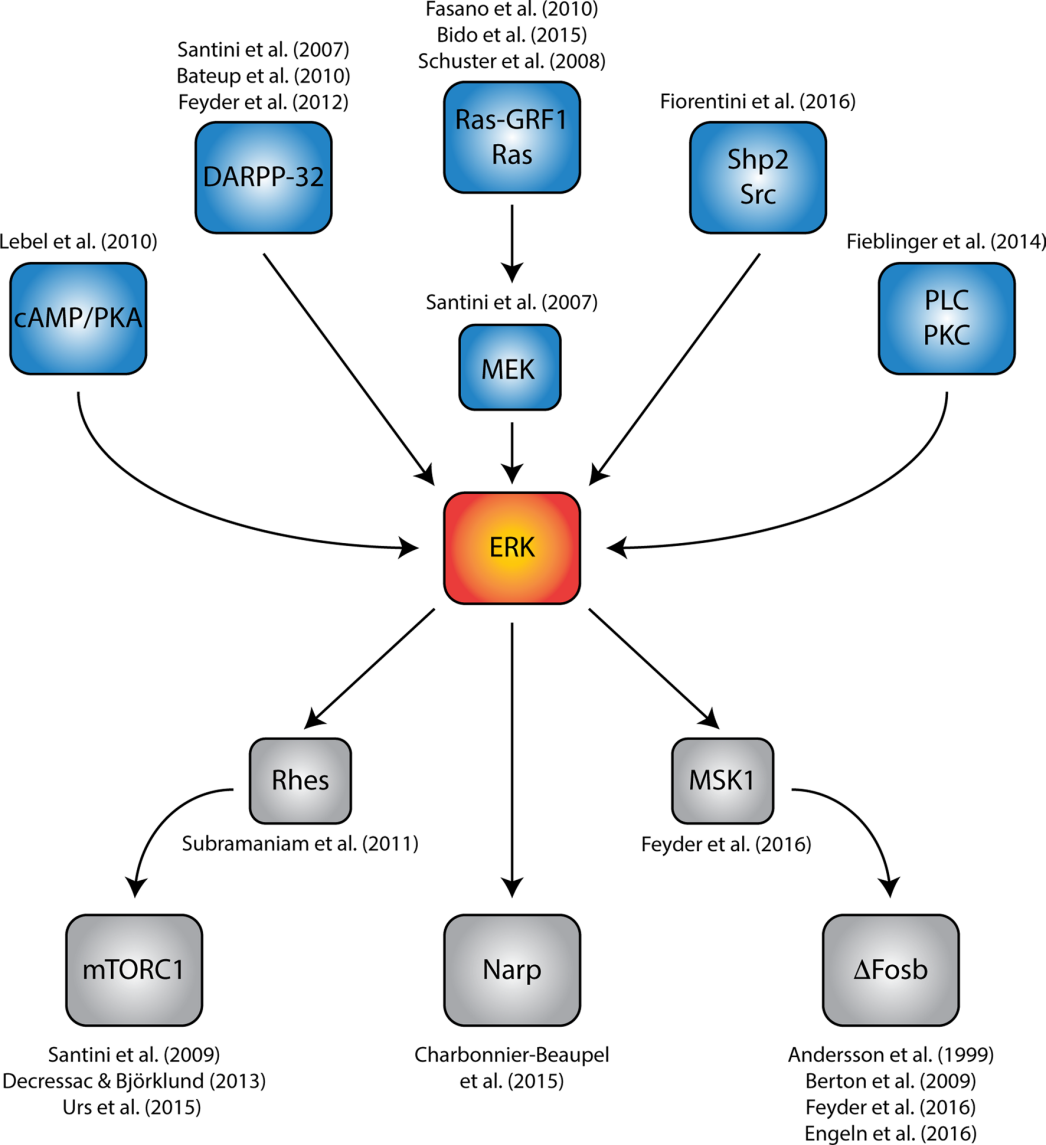
Diagram summarizing the central role played by abnormal ERK signaling in LID. Hyperactivation of ERK in response to stimulation of sensitized dopamine D1 receptors is emerging as a central point of convergence for several signaling pathways implicated in dyskinesia, including the cAMP/PKA/DARPP-32, the Ras-GRF1/Ras/MEK, the phospholipase C (PLC)/protein kinase C (PKC) and the Shp2/Src cascades. Pharmacological or genetic inhibition of various constituents of these intracellular pathways has been shown to reduce LID. Activation of ERK generates additional pathological responses, such as abnormal Rhes/mTORC1 signaling, and overexpression of ∆FosB (mediated by MSK1) and Narp. Several interventions counteracting these changes have been shown to reduce dyskinesia

The hyperactivation of cAMP/PKA/DARPP-32 produced by l-DOPA in the dopamine-depleted striatum results in the dysregulation of multiple signaling pathways ultimately implicated in dyskinesia. Among these downstream cascades, the extracellular signal-regulated kinases 1/2 (ERK) pathway, plays a particularly relevant role and has been the subject of intense investigation (Fig. 2). Gerfen et al. (2002) showed that the loss of dopamine input to the striatum results in a large increase in ERK phosphorylation, in response to administration of a D1R agonist. A few years later Pavon et al. (2006) reported that a similar effect occurred also in response to administration of l-DOPA and in association with LID. The increase in ERK phosphorylation induced by l-DOPA was then localized to dSPN and shown to occur in response to activation of D1R (Darmopil et al. 2009; Santini et al. 2009a; Westin et al. 2007).

In neuronal cells, ERK activity is modulated by exchange factors, such as the Ras-guanyl nucleotide releasing factor 1 (Ras-GRF1) and the calcium- and diacylglycerol-regulated guanine nucleotide exchange factor II (CalDAG-GEFII), which is particularly abundant in SPN. Calcium-dependent activation of Ras-GRF1 and CalDAG-GEFII induces the exchange of GDP for GTP on the small G-protein Ras (Farnsworth et al. 1995; Toki et al. 2001), which in turn stimulates the protein kinase Raf. Raf phosphorylates the mitogen-activated protein kinase/ERK kinase (MEK), ultimately leading to phosphorylation/activation of ERK.

The first demonstration of the involvement of D1R-dependent activation of ERK in LID was obtained in a mouse model of PD using SL327, a systemically deliverable MEK inhibitor (Santini et al. 2007). Subsequent studies further corroborated the importance of ERK in dyskinesia. For instance, reduced ERK activation and concomitant attenuation of LID were achieved with lovastatin (Schuster et al. 2008), an inhibitor of Ras (Li et al. 2005b). Genetic inactivation or lentiviral-mediated downregulation of Ras-GRF1 resulted in the concomitant reduction of l-DOPA-induced phosphorylation of ERK and dyskinesia (Fasano et al. 2010; Bido et al. 2015). Furthermore, increased expression of CalDAG-GEFII, potentially linked to abnormal activation of ERK, was found in the striata of dyskinetic mice (Crittenden et al. 2009).

The increase in ERK phosphorylation produced by D1R requires intact PKA activity and phosphorylation of DARPP-32 at Thr34 (Lebel et al. 2010; Santini et al. 2007, 2012); but see also (Gerfen et al. 2008). Based on these observations, it was suggested that the inhibition of PP-1 exerted by DARPP-32 would decrease the activity of the striatal-enriched protein tyrosine phosphatase (STEP), thereby counteracting the dephosphorylation of ERK (Paul et al. 2003; Valjent et al. 2005). Additional work showed that the increase in ERK phosphorylation depends also on the interaction of D1R with the Src homology 2 domain-containing phosphatase 2 (Shp2) (Fiorentini et al. 2011, 2013). In particular, it was proposed that D1R-mediated activation of PKA leads to stimulation of Shp2. This, in turn, would disinhibit Src kinases, which positively regulate the Ras/Raf/ERK signaling cascade (Fiorentini et al. 2011; Yang et al. 2006). In accordance with this possibility, persistent Shp2/ERK activation was found in the striata of hemi-parkinsonian rats displaying dyskinetic behavior in response to l-DOPA or a D1R agonist (Fiorentini et al. 2013). Furthermore, lentiviral-mediated downregulation of Shp2 decreased ERK phosphorylation and attenuated LID (Fiorentini et al. 2016).

The increase in ERK activity mediated by l-DOPA or a D1R agonist is reduced by pharmacological inhibition of the mGlu5R, an intervention which also attenuates dyskinesia (Rylander et al. 2009). The mGlu5R-dependent stimulation of ERK is likely to occur via Gαq protein-mediated activation of phospholipase C and calcium-dependent signaling (Fieblinger et al. 2014; Rylander et al. 2009), thereby suggesting a further potential mechanism underlying the pathological activation of ERK in LID.

Studies performed in various models of PD indicate that the abnormal regulation of ERK observed in SPN subsides during the course of chronic l-DOPA administration, suggesting its primary involvement in the development, rather than in the maintenance of dyskinesia (Ding et al. 2011; Santini et al. 2010). Interestingly, this normalization is paralleled by increased ERK phosphorylation in striatal cholinergic interneurons (Chl), which may, therefore, represent an additional neural substrate specifically implicated in the long-term manifestation of dyskinesia (Ding et al. 2011). In line with this possibility, diphtheria toxin-mediated ablation of Chl attenuates LID, without affecting the anti-akinetic properties of l-DOPA (Won et al. 2014). The increase in ERK activity observed in Chl is indicative of increased excitability, which may be causally related to LID. This is supported by the observation that, in a mouse model of PD, short-pulse optogenetic stimulation of Chl exacerbates the dyskinetic response to l-DOPA (Bordia et al. 2016). Conversely, in the same PD model, LID is reduced by interventions that counteract Chl hyper-excitability by antagonizing excitatory histamine H2 receptors, whose activity is pathologically enhanced in the Chl of dyskinetic mice (Lim et al. 2015).

Activation of ERK results in the stimulation of complex intracellular cascades, involving distinct signaling pathways linked to the regulation of mRNA translation and gene transcription. A large body of evidence indicates that these downstream events represent an essential causal factor in the emergence of LID and several components of these intracellular pathways have been identified as potential targets for anti-dyskinetic interventions (Fig. 2).

## Involvement of the mammalian target of rapamycin complex 1 in LID

One interesting downstream effector target of ERK is the mammalian target of rapamycin complex 1 (mTORC1) signaling cascade, which is involved in the regulation of protein synthesis. mTORC1 is activated by the small G proteins, Ras homolog enriched in brain (Rheb) and Ras homolog enriched in striatum (Rhes) (Long et al. 2005; Subramaniam et al. 2011), which are inhibited by the GTPase-activating heterodimer composed of tuberous sclerosis (TSC) 1 and 2 (Laplante and Sabatini 2012). ERK inhibits the TSC1/TSC2 complex, thereby promoting mTORC1 activity (Ma et al. 2005; Roux et al. 2004; Tsokas et al. 2007). This results in the control of downstream effectors, including the p70 ribosomal S6 kinases (Ruvinsky and Meyuhas 2006; Thomas et al. 1979) and the initiation factor 4E-binding protein 1 (Gingras et al. 2001), leading to enhanced mRNA translation (Laplante and Sabatini 2012). Augmented signaling along the mTORC1 cascade has been described in rodent models of dyskinesia (Decressac and Bjorklund 2013; Santini et al. 2009b; Subramaniam et al. 2011). Importantly, administration of mTORC1 inhibitors, such as rapamycin, or its analog temsirolimus (CCI-779), was shown to reduce the development and expression of LID in rodent models of PD (Decressac and Bjorklund 2013; Santini et al. 2009b; Urs et al. 2015). The importance of abnormal mTORC1 activation in dyskinesia is further substantiated by the observation that LID is reduced in Rhes-deficient mice (Subramaniam et al. 2011).

## Abnormal gene transcription in LID

Upon activation, a large proportion of ERK translocates to the nucleus (Chen et al. 1992), where it controls a number of effectors implicated in various aspects of transcriptional regulation (Treisman 1996; Whitmarsh 2007). One major group of ERK downstream targets is represented by early response transcription factors, among which FosB has attracted considerable interest with regard to its involvement in LID. Chronic administration of l-DOPA induces a progressive accumulation of stable isoforms of FosB, collectively denominated ∆FosB, in the striata of animal models of LID and dyskinetic patients (Andersson et al. 1999; Berton et al. 2009; Darmopil et al. 2009; Fasano et al. 2010; Feyder et al. 2016; Lindgren et al. 2011; Pavon et al. 2006; Tekumalla et al. 2001). The increase in ∆FosB associated with dyskinesia is limited to the D1R-expressing dSPN and is mediated by activation of cAMP/PKA and ERK (Andersson et al. 1999; Darmopil et al. 2009; Feyder et al. 2016; Fasano et al. 2010; Lebel et al. 2010; Schuster et al. 2008; Chen et al. 2017).

∆FosB accumulation plays a causal role in LID. Initial experiments showing that antisense oligonucleotides against *fosB* reduced dyskinesia were followed by several studies in which LID was exacerbated or reduced by modulating FosB-dependent transcriptional activity. ∆FosB forms heterodimers with members of the *jun* family of transcription factors (typically JunD), which promote the expression of late response genes by binding to their activator protein-1 (AP-1) consensus sites (Hope et al. 1994). Striatal overexpression of a truncated form of JunD (∆JunD), lacking the transactivation domain and acting as a ∆FosB dominant-negative, reduced LID in the MPTP non-human primate model of PD (Berton et al. 2009). In contrast, overexpression of ∆FosB in the dopamine-depleted striatum of rats with a unilateral 6-OHDA lesion was sufficient to elicit abnormal involuntary movements indicative of dyskinetic behavior (Cao et al. 2010). In another study, selective silencing of ∆FosB expressing SPN, using the Daun02 inactivation method (Ghosh et al. 2000), was found to decrease the severity of LID (Engeln et al. 2016).

Work in the 6-OHDA mouse model of PD showed that overexpression of ∆FosB induced specifically in dSPN exacerbates LID. Conversely, dyskinesia is reduced by overexpression of ∆cJun, a truncated form of cJun lacking transcriptional activity (Feyder et al. 2016). The ability of ∆cJun to reduce LID suggests a possible involvement of dysregulated cJun in dyskinesia. This idea is supported by recent work showing that administration of l-DOPA to mice with a 6-OHDA lesion increases cJun phosphorylation (Spigolon et al. 2018). This effect occurs selectively in dSPN and is linked to increased strength at corticostriatal synapses, which has been associated with LID (Calabresi et al. 2016; Spigolon et al. 2018).

∆FosB expression and LID are modulated by genetic manipulation of the downstream regulatory element antagonistic modulator (DREAM). DREAM is activated by calcium and cAMP, and functions as a transcriptional repressor by binding to a regulatory element located downstream of the transcription initiation site of several genes, including members of the *fos* family. In a mouse model of PD, DREAM deficiency exacerbates the accumulation of ∆FosB and the severity of LID, whereas overexpression of a dominant-active DREAM mutant reduces both ∆FosB expression and dyskinesia (Ruiz-DeDiego et al. 2015a).

LID is also associated with abnormal expression of the transcription factor Zif268 (also called NGFI-A, Krox24, or Egr1) (Bastide et al. 2014; Carta et al. 2005; Fisone 2010; Carta et al. 2010). This effect may depend on concomitant activation of ERK, which mediates the increase in *zif268* expression produced by cocaine in the striatum (Valjent et al. 2006). In the rat model of PD, repeated administration of l-DOPA produces a persistent elevation of *zif268* mRNA expression in dSPN (Carta et al. 2005). Increased Zif268 may in turn lead to augmented expression of the activity-regulated cytoskeleton-associated protein (Arc, or Arg3.1) (Li et al. 2005a), which is also up-regulated in the dSPN of dyskinetic rats (Sgambato-Faure et al. 2005).

Aberrant regulation of ∆FosB, Zif268 and Arc is likely to contribute to the changes in synaptic plasticity described in experimental models of LID. In fact, these transcription factors regulate LTP and synaptic morphology (Guzowski et al. 2000; Jones et al. 2001; Nestler et al. 2001; Peebles et al. 2010; Plath et al. 2006), which undergo profound changes in dyskinesia. For instance, they could be implicated in the impairment of synaptic downscaling proposed to mediate dyskinetic behavior (Calabresi et al. 2016).

Dopamine depletion is associated with a dramatic loss of synaptic spines in dSPN and iSPN (Day et al. 2006; Suarez et al. 2014, 2016). Administration of l-DOPA re-establishes normal spine density in D2R expressing iSPN (Suarez et al. 2014, 2016). However, these newly formed synapses display thinner neck diameter and smaller PSD. Consequently, their numeric recovery does not rescue overall synaptic strength (Suarez et al. 2016). In contrast, l-DOPA normalizes synaptic strength in D1R-expressing dSPN, in spite of persistent reduction in spine density. This paradoxical effect has been ascribed to increased spine volume and PSD length (Suarez et al. 2016), possibly caused by the parallel increase in ∆FosB, Zif268 and Arc in dSPN.

A number of studies have provided additional information concerning the involvement of abnormal gene expression in LID. Analysis based on DNA microarray technology revealed major differences in striatal gene expression between dyskinetic and non-dyskinetic rats, both receiving l-DOPA. In particular, dyskinesia was associated with up-regulation of genes involved in calcium homeostasis, synaptic activity and remodeling, and downregulation of genes related to energy metabolism, or encoding ribosomal proteins (Konradi et al. 2004).

Using a similar approach, gene expression changes associated with de novo or long-term administration of l-DOPA were compared in dopamine-depleted rats. The overall transcriptomic response was higher following chronic l-DOPA treatment. Moreover, distinct clusters of genes were specifically deregulated in the two conditions, with only a small set of genes (including *arc*) affected by both treatments (El Atifi-Borel et al. 2009). Interestingly, genes involved in signal transduction and synaptic transmission were up-regulated by acute treatment and potentially involved in the initial response necessary for the development of LID. Other genes related to structural and cellular alterations were instead up-regulated by chronic treatment and possibly necessary for the persistent modifications involved in the maintenance of dyskinesia (El Atifi-Borel et al. 2009).

Recent work focused on the identification of early changes in gene expression implicated in the development of dyskinesia. A time course of the effect of l-DOPA in the 6-OHDA mouse model of PD identified different sets of genes deregulated at 1 h (immediate early genes, mitogen-activated protein kinase signaling-related and synapse-associated genes), 3 h (long-term depression-related genes) and 6 h (genes regulating protein phosphatase activity) (Charbonnier-Beaupel et al. 2015). Among these changes, those requiring ERK activation were selected as more likely molecular markers involved in the early development of LID. The study then focused on the immediate early gene, *nptx2*, whose overexpression was found to be ERK-dependent and associated with a high dyskinetic response (Charbonnier-Beaupel et al. 2015). *Nptx2* codes for the neuronal activity-regulated pentraxin (Narp), which is involved in synaptic plasticity (Xu et al. 2003) and may represent a potential target for anti-dyskinetic therapy. Accordingly, Narp knockout mice showed a reduction of dyskinesia, while virus-mediated overexpression of *Nptx2* worsened LID (Charbonnier-Beaupel et al. 2015).

A cell-type specific analysis of gene expression changes in dSPNs and iSPNs following chronic l-DOPA was provided by Heiman et al. (2014) using the translating ribosome affinity purification (TRAP) method. Dopamine depletion alone induced gene alterations in both classes of SPNs. However, chronic l-DOPA treatment strongly affected the expression profile in dSPNs, but induced only small changes in iSPNs. Besides increased levels of mitogen-activated protein kinase-related genes, dSPN showed up-regulation of several markers involved in homeostatic processes (e.g. *Rgs6, Sstr2, Kcnn3*), which were most likely activated to counteract abnormal D1R transmission. A noteworthy finding observed exclusively in dSPNs was the correlation between expression of genes such as *Nurr1*, *Itch*, *Fra*-*1* and *Dusp* and the severity of the dyskinetic response to l-DOPA (Heiman et al. 2014).

## Chromatin remodeling in LID

**Fig. 3:**
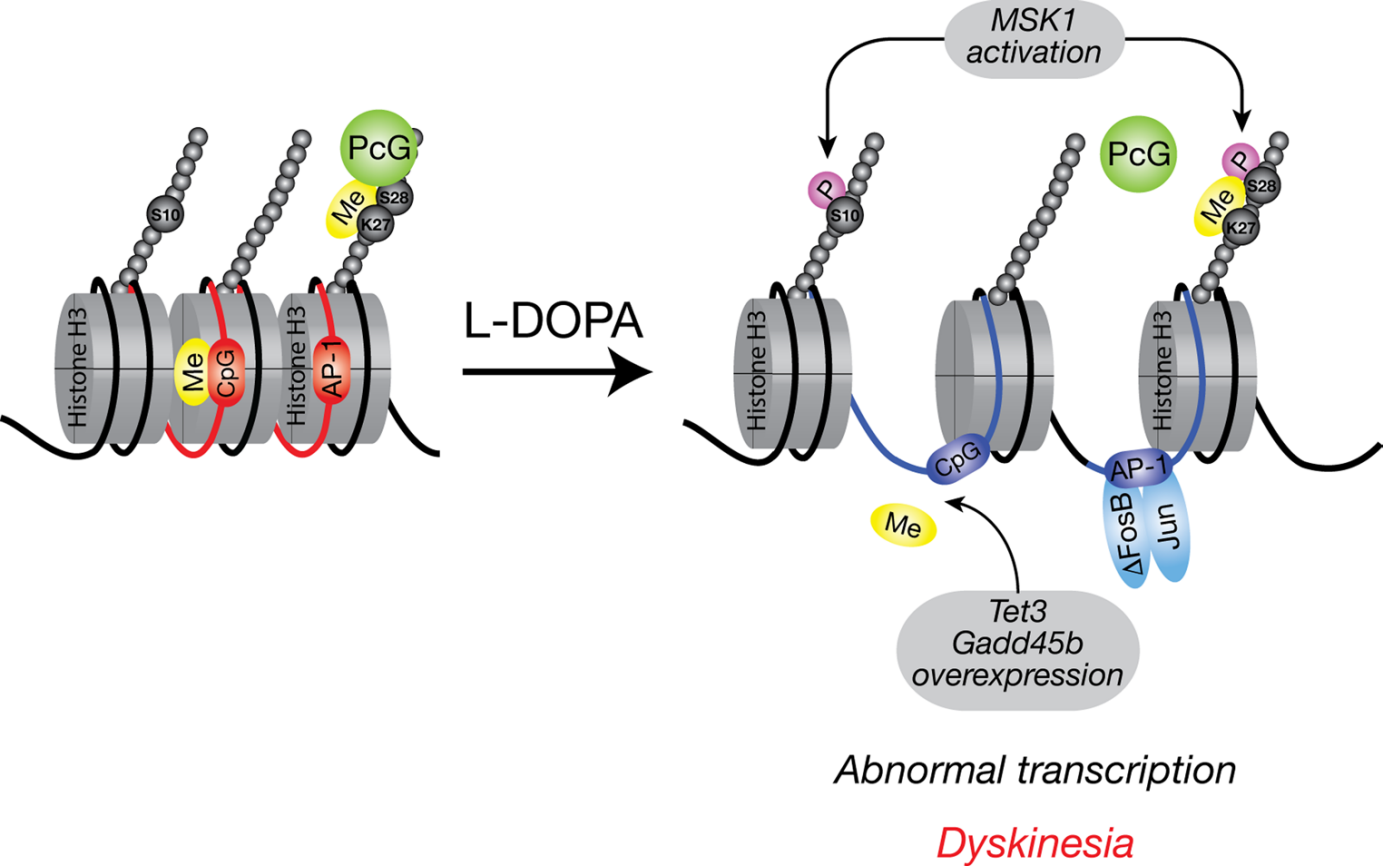
Summary of the main chromatin modifications associated with LID. During dyskinesia, ERK-mediated activation of mitogen- and stress-activated kinase 1 (MSK1) results in the phosphorylation of histone H3 at Ser10 and 28. Both modifications promote gene transcription. Enrichment of histone H3 phosphorylated at Ser10 has been reported to occur at the *fosB* promoter and may, therefore, participate in the accumulation of ∆FosB associated with LID. ∆FosB forms heterodimers with members of the *jun* family of transcription factors which bind to the activator protein-1 (AP-1) sites of late response genes to regulate their transcription. Phosphorylation of histone H3 at Ser28 counteracts the action of Polycomb group (PcG) proteins, which suppress gene transcription via methylation at Lys27. Enrichment of Ser28 phosphorylation, which leads to displacement of PcG, has been found at the promoters of several genes up-regulated in the striata of dyskinetic mice. LID is also accompanied by increased expression of DNA demethylases, such as Tet3 and Gadd45b. This regulation is paralleled by reduced methylation of CpG dense regions of DNA, which is indicative of increased transcriptional activity

The abnormal D1R signaling produced by the combination of dopamine depletion and chronic administration of l-DOPA results in a number of chromatin modifications associated with dyskinesia (Fig. 3). Activation of mitogen-activated protein kinases, including ERK, results in the phosphorylation of histone H3 at Ser10 and Ser28, which mediates the rapid induction of *c*-*jun* and *c*-*fos* (Clayton and Mahadevan 2003; Heiman et al. 2014). In LID, the hyperactivation of cAMP and ERK signaling is accompanied by a similar response, which requires D1R and occurs selectively in dSPN (Feyder et al. 2016; Sodersten et al. 2014; Santini et al. 2007, 2012; Darmopil et al. 2009; Ruiz-DeDiego et al. 2015a).

Studies in the mouse showed that the increase in ∆FosB implicated in LID depends on the mitogen- and stress-activated kinase 1 (MSK1), a major downstream target of ERK. Chromatin immunoprecipitation (ChIP) analysis showed that ERK-mediated activation of MSK1 leads to the accumulation of histone H3 phosphorylated on Ser10, at the promoter of *fosB* (Feyder et al. 2016). These findings provide a possible mechanism explaining the ability of sensitized D1R to promote ∆FosB expression. In line with the involvement of MSK1 in the regulation of ∆FosB, MSK1 knockout mice display attenuated dyskinesia in response to l-DOPA (Feyder et al. 2016) but see also (Alcacer et al. 2014).

The abnormal activation of PKA/DARPP-32 and ERK/MSK1 caused by l-DOPA in experimental parkinsonism is also leading to hyper-phosphorylation of histone H3 at Ser28 (Sodersten et al. 2014). This modification counteracts the repressive action exerted on transcription by Polycomb group (PcG) proteins (Gehani et al. 2010). Polycomb repressive complex 2 (PRC2) catalyzes di- and trimethylation of Lys27 on histone H3 at the promoters of specific genes, thereby establishing repression. In dyskinesia, DARPP-32- and MSK1-mediated phosphorylation at Ser28 leads to dissociation of PcG and de-repression of gene transcription (Sodersten et al. 2014). Thus, analysis of data from RNA- and ChIP-sequencing shows that induction of Ser28 phosphorylation on histone H3 correlates with genome-wide increase of mRNA transcripts from loci marked by Lys27 trimethylation (Sodersten et al. 2014).

Among the up-regulated genes positive for histone H3 Ser28 phosphorylation and Lys27 trimethylation were transcription factors involved in dopamine-mediated responses and potentially implicated in LID, such as *atf3* and *npas4* (Sodersten et al. 2014). Increased *atf3* expression in the dorsal striatum is also triggered by chronic administration of amphetamine, a drug which augments dopamine release and produces a strong motor stimulant effect (Green et al. 2008). Npas4 is also interesting in the context of LID, since it promotes the expression of other immediate early genes associated with dyskinesia, such as *arc*, *zif268* and *c*-*fos* (Ramamoorthi et al. 2011).

A particularly stable form of chromatin remodeling is produced by the methylation of DNA within short interspersed sequences enriched in cytosine and guanine, called CpG islands. Methylation of cytosines within CpG islands, which are thought to represent transcription initiation sites, is typically associated with silencing of gene expression (Deaton and Bird 2011). Regulation of DNA methylation has been intensively investigated with regard to its involvement in memory, addictive behavior and synaptic plasticity (Halder et al. 2016; LaPlant et al. 2010; Levenson et al. 2006; Miller et al. 2008) and recent studies provide information concerning its impact on LID. Chronic administration of l-DOPA in combination with dopamine depletion results in up-regulation of two DNA demethylases, Tet3 and Gadd45b, in the striata of dyskinetic rodents (Figge et al. 2016; Park et al. 2016). In line with this finding, genome-wide analysis of methylation identified a subset of CpG-enriched sequences, in which changes in methylation occurred in response to both dopamine depletion and administration of l-DOPA. This subset was enriched in transcripts aberrantly transcribed in the D1R expressing dSPN (Heiman et al. 2014), indicating a correlation between decreased DNA methylation and activation of genes associated with LID, including *fosB* and *arc* (Figge et al. 2016). Remarkably, the CpG-enriched sequences undergoing changes in methylation were mainly located in regulatory regions at long distance from the promoters (Gray et al. 2015) and implicated in activity-dependent regulation of immediate early genes, including *fos* (Malik et al. 2014).

The aforementioned findings suggest that interventions which control abnormal gene activation by promoting DNA methylation may represent potential strategies for the treatment of LID (Figge et al. 2016). Thus, administration of methionine, which enhances DNA methylation, reduces LID in rats, whereas RG-108, a DNA methyltransferase inhibitor, exacerbates this condition (Figge et al. 2016). However, genetic inactivation of Gadd45b, an intervention that should reduce DNA demethylation, has been found to enhance LID (Park et al. 2016). In contrast, increased expression of Gadd45b, which should decrease DNA methylation, thereby aggravating abnormal gene expression, reduces dyskinesia (Park et al. 2016). Therefore, the dynamics of the regulation of DNA methylation during LID, as well as the identification of specific targets for the development of effective therapeutic strategies, remain to be fully defined.

## Conclusions

The use of animal models has been essential to discover numerous signaling abnormalities potentially implicated in LID, thereby providing useful information for the generation of new strategies to efficiently control the motor symptoms of PD. Drugs targeting various components of the Ras-ERK and mTORC1 signaling cascades are currently used, or under clinical investigation as anti-tumoral agents. Inhibitors of Raf, such as sorafenib, vemurafenib and dabrafenib, and MEK 1/2, such as trametinib, have been approved for the treatment of various types of cancers. Additional compounds with a similar pharmacological profile have entered clinical trials (Mandal et al. 2016). Different classes of mTOR inhibitors, including analogs of rapamycin (e.g. everolimus) and ATP-competitive inhibitors (AZD2014), have also been approved, or are in clinical trials as anticancer therapies (Xie et al. 2016).

The relative clinical safety of these compounds represents an encouraging feature with regard to their possible use for the treatment of neurodegenerative and neuropsychiatric diseases. However, the use of these drugs is still complicated by the risk of negative side effects, since they must be administered chronically and at relatively high doses. This problem could be circumvented combining low doses of drugs acting on distinct targets. The resulting cumulative effect exerted by partial inhibition of multiple signaling pathways may be sufficient to counteract LID, without abolishing intracellular processes necessary for normal cell function. In support of this approach, various combinations of Raf, MEK1/2 and mTORC1 inhibitors are commonly used in cancer treatment (Mandal et al. 2016).

An additional way to minimize the risk of complications is to identify effector targets located downstream of critical elements implicated in LID. This approach is exemplified by the search for signaling pathways (e.g. mTORC1), or genes (e.g. ∆FosB and Narp) controlled by ERK, whose dysregulation is ultimately implicated in dyskinesia. By the same reasoning, it will be important to distinguish specific constituents of the chromatin remodeling machinery, particularly enzymes catalyzing covalent modifications of both histones and DNA, which may represent viable druggable targets.

Progress in the understanding of the mechanisms of LID has also led to the identification of several molecular events, such as activation of key signaling components and overexpression of specific genes, which can be utilized to evaluate the potential efficacy of various types of interventions. For instance, continuous, as opposed to pulsatile, administration of l-DOPA, which significantly reduces the risk of developing LID, is not associated with increased phosphorylation of DARPP-32 and ERK, or with accumulation of ∆FosB in striatal neurons (Lebel et al. 2010). Similarly, administration of antagonists at mGlu5R, which attenuates dyskinesia in experimental PD, is associated with reduced striatal levels of phosphorylated ERK and MSK1 (Rylander et al. 2009). Altogether, these biochemical markers represent important tools for the rapid assessment of putative anti-dyskinetic therapies.
